# IgE sensitisation to cow’s milk proteins among children with suspected cow’s milk allergy: A retrospective study in Abidjan^☆^

**DOI:** 10.1016/j.waojou.2026.101375

**Published:** 2026-04-02

**Authors:** Amah Patricia Victorine Goran-Kouacou, Adjoumanvoulé Honoré Adou, Yida Jocelyne Séri, Oppong Richard Yéboah, Adisa Mondah, Aya Ursule Aniela Assi, Nicole Ingrid Niamba, Koffi N'Guessan, Kouabla Liliane Siransy, Séry Romuald Dassé

**Affiliations:** a Department of Immunology and Allergology, Faculty of Medicine, Félix Houphouët-Boigny University, Abidjan, Republic of Côte d'Ivoire; b Immunology and Allergology Laboratory, University Hospital of Cocody, Abidjan, Republic of Côte d'Ivoire; c Faculty of Medicine, Félix Houphouët-Boigny University, Abidjan, Republic of Côte d'Ivoire

**Keywords:** Food allergy, Child, Cow's milk proteins, IgE sensitisation, Skin prick test

## Abstract

**Introduction:**

Cow's milk protein allergy (CMPA) is the most common food allergy in infants. In low- and middle-income countries, limited access to oral food challenges makes the skin prick test (SPT) a practical alternative for identifying IgE-mediated forms.

**Aim:**

To determine the prevalence of IgE sensitisation to cow's milk proteins among children referred for suspected CMPA and to identify associated clinical factors.

**Methods:**

A retrospective analytical study was conducted from January 2021 to December 2024 in the Immunoallergology Unit of the University Hospital of Cocody, Abidjan, Côte d’Ivoire. Children aged 0–36 months underwent SPT using a commercial extract or a prick-to-prick technique with pasteurised/UHT milk. A wheal ≥3 mm above the negative control defined positivity. Clinical data included age, feeding mode, family atopy, and symptoms type. Cutaneous manifestations were analysed as a composite variable due to inconsistent distinction between eczema and urticaria. Proportions were expressed with 95% confidence intervals (Wilson method), and group comparisons used chi-square or Fisher's exact tests (p < 0.05).

**Results:**

Two hundred children were included (mean age 17.8 months; 55.5% boys). Overall, 29.5% (59/200; 95% CI 23.6–36.2) had a positive SPT. Sensitisation was higher in infants <12 months (38.1% vs 23.3%, p = 0.021), in those with eczema (37.8% vs 17.3%, p < 0.001), and in those with a family history of atopy (32.0% vs 9.1%, p = 0.039). Digestive symptoms were inversely associated with sensitisation (14.9% vs 36.8%, p = 0.001). No cases of anaphylaxis were documented.

**Conclusion:**

Nearly one-third of children referred for suspected CMPA showed IgE sensitisation. In resource-limited settings, the SPT remains a useful triage tool, particularly in infants with eczema or family atopy, whereas isolated digestive symptoms are less predictive. Interpretation should consider the retrospective design and the absence of confirmatory oral food challenges.

## Introduction

Cow's milk protein allergy (CMPA) is the most frequent food allergy in infants and young children.^1^ It includes both immunoglobulin E (IgE)-mediated and non-IgE-mediated forms. The IgE-mediated type, the most frequent and the only one detectable by skin testing, usually resolves spontaneously in most children but can persist beyond early childhood in some cases.^2^ In the absence of standardised diagnostic protocols, prolonged dietary exclusions may delay food diversification, impair growth, and increase financial burden on families, highlighting the need for an evidence-based diagnostic approach from the first consultation.^3^^,^^4^ In low- and middle-income countries, access to specialised allergy investigations, particularly the oral food challenge (OFC), remains limited. In this context, the skin prick test (SPT) provides a simple, inexpensive, and reproducible method for assessing IgE-mediated sensitisation to cow's milk proteins.^5^, ^6^, ^7^ International guidelines emphasise that a positive SPT indicates sensitisation rather than confirmed clinical allergy, with the OFC remaining the gold standard when feasible.^6^^,^^7^ A diagnostic pathway centred on SPT results, interpreted alongside clinical history and symptom patterns, therefore offers a pragmatic approach to identifying IgE-mediated forms and rationalising dietary exclusion or reintroduction decisions. Available data from sub-Saharan Africa remain scarce but suggest a non-negligible prevalence of cow's milk protein sensitisation among infants and children referred for suspected food allergy.^8^ Furthermore, longitudinal studies show that tolerance is usually acquired before the age of 3 years, while certain IgE-mediated forms persist and require ongoing follow-up.^2^^,^^9^ In many low-resource settings, access to hypoallergenic formulas and complementary diagnostic tools such as specific IgE assays or oral food challenges remains limited.^7^ These constraints influence both diagnostic pathways and nutritional management, reinforcing the need to assess the performance of the SPT as an initial triage tool. Within this context, we conducted a retrospective evaluation in the University Hospital of Cocody, Abidjan, Côte d’Ivoire to assess the role of the skin prick test in diagnosing cow's milk protein allergy. The objective was to estimate the prevalence of IgE-mediated sensitisation to cow's milk proteins among children referred for suspected CMPA and to identify the main clinical profiles associated with a positive SPT.

## Materials and methods

### Study design and setting

This retrospective analytical study was conducted from January 2021 to December 2024 in the Immunoallergology Unit of the University Hospital of Cocody, Abidjan, Côte d’Ivoire. The Immunoallergology Unit provides consultations for both children and adults and performs allergological investigations.

### Study population

The analysis focused on medical records of children aged 0–36 months evaluated for suspected CMPA. Participants were either referred by paediatricians for allergological testing or seen directly in consultation at the Allergy Unit. Each child was included based on the initial allergological evaluation documented in the medical record. Possible repeated SPTs performed at a later age were not analysed in this retrospective design.

### Eligibility criteria

Eligible participants were children who had undergone a SPT for cow's milk proteins as part of an allergy assessment, with complete clinical information including evocative signs (digestive, cutaneous, or respiratory) and relevant family history. Incomplete records (missing clinical data or test results) and cases where the SPT had been performed for allergens other than cow's milk proteins were excluded.

### Data collection

Information was systematically extracted from medical records, including age, sex, feeding pattern (exclusive breastfeeding, mixed feeding, or formula feeding), symptom type (cutaneous, digestive, or respiratory) related to milk ingestion, first-degree family history of atopy, and SPT result to cow's milk proteins. Information regarding the use of nutritional substitutes (soy-based, rice-based or locally available preparations) was not consistently documented in the medical records and could not be analysed.

### Skin prick testing

SPTs were performed in the Allergy Unit in accordance with international paediatric allergy recommendations. When available, a commercial extract was used; otherwise, a prick-to-prick technique with whole pasteurised or UHT cow's milk was performed.^10^^,^^11^ A positive control (histamine 10 mg/mL) and a negative control (saline solution) were systematically applied. Readings were taken after 15–20 min. A mean wheal diameter ≥3 mm larger than the negative control defined positivity. Test validity was confirmed by a visible response to the histamine control. The choice between commercial extract and prick-to-prick technique depended on reagent availability. A comparative analysis of SPT results according to the method used was not feasible within this retrospective dataset.

### Variables and outcome measures

The primary outcome was SPT positivity to cow's milk proteins. Prespecified clinical factors of interest included age (<12 vs ≥ 12 months), presence of eczema, gastrointestinal or respiratory symptoms, family history of atopy, and feeding mode (exclusive or mixed formula feeding). Cutaneous manifestations were analysed as a single composite variable (“cutaneous signs”), as the distinction between eczema and urticaria was not consistently specified in the medical records.

### Statistical analysis

Categorical variables were expressed as counts and percentages. Proportions (overall and stratified) were reported with 95% confidence intervals using the Wilson method. Between-group comparisons were performed using Pearson's chi-square or Fisher's exact test, as appropriate. A two-sided p-value <0.05 was considered statistically significant.

## Results

### General characteristics

A total of 200 children aged 0–36 months were included. The mean age was 17.8 ± 11.9 months (95% CI: 16.1–19.5), with a male-to-female ratio of 1.25. Most children were exclusively formula-fed (75%), and a family history of atopy was reported in 89%. Cutaneous manifestations were predominant (59.5%), followed by digestive (33.5%) and respiratory (7%) symptoms (Table 1). No cases of documented anaphylaxis were identified in the available medical records.

**Table 1:** Sociodemographic, nutritional and clinical characteristics of the 200 children included in the study

Parameter	n (=200)	%
Boys	111	55.5
Girls	89	44.5
Age 1–11 months	84	42.0
Age 12–23 months	72	36.0
Age 24–36 months	44	22.0
Mean ± SD age (months)	17.8 ± 11.9	95% CI 16.1–19.5
Exclusive formula feeding	150	75.0
Mixed feeding	50	25.0
Cutaneous signs (eczema/urticaria)	119	59.5
Digestive symptoms (vomiting/diarrhoea)	67	33.5
Respiratory symptoms (rhinitis/wheezing)	14	7.0
Family history of atopy (first-degree)	178	89.0

### Skin prick test results

**Fig. 1:**
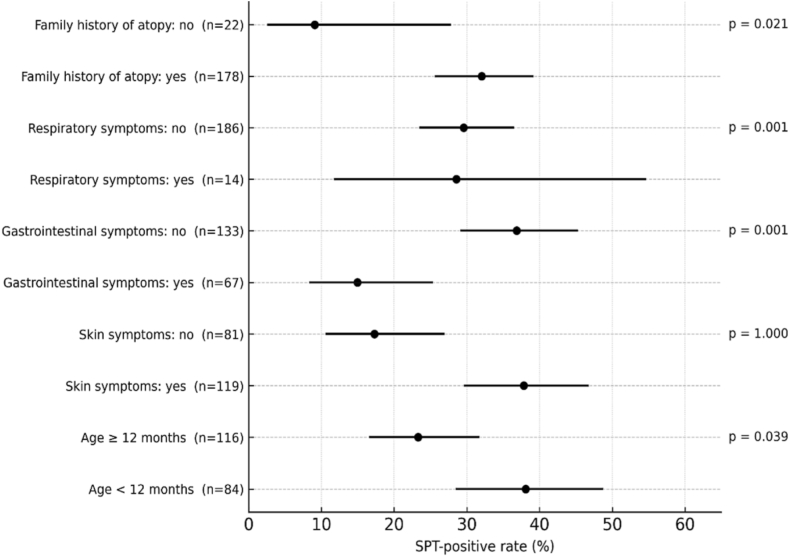
Proportion of positive SPT results (%) and 95% confidence intervals (Wilson method) according to clinical and demographic characteristics. Dots represent the proportion of children with a positive skin prick test for each category. Horizontal bars indicate 95% confidence intervals. P-values were derived from Pearson's chi-square or Fisher's exact test, depending on expected cell counts

The SPT to cow's milk proteins was positive in 59 children (29.5%; 95% CI: 23.6–36.2). IgE sensitisation was significantly more frequent in infants younger than 12 months (38.1% vs 23.3% beyond 12 months, p = 0.021), in those with eczema (37.8% vs 17.3%, p < 0.001), and in those with a family history of atopy (32.0% vs 9.1%, p = 0.039). Conversely, positivity was less common in children with digestive symptoms (14.9% vs 36.8%, p = 0.001). No association was observed with respiratory manifestations (p = 1.00). Detailed distributions are shown in Table 2 and illustrated in Fig. 1.

**Table 2:** Proportion of positive skin prick tests (SPT+) according to clinical and demographic characteristics.

Factor	Category	SPT+ (n)	SPT– (n)	Total	SPT+% [95% CI]	p-value
Age	<12 months	32	52	84	38.1 [28.4–48.8]	0.021^a^
	≥12 months	27	89	116	23.3 [16.5–31.7]	
Cutaneous signs	Yes	45	74	119	37.8 [29.6–46.8]	<0.001
	No	14	67	81	17.3 [10.6–26.9]	
Digestive symptoms	Yes	10	57	67	14.9 [8.3–25.3]	0.001
	No	49	84	133	36.8 [29.1–45.3]	
Respiratory symptoms	Yes	4	10	14	28.6 [11.7–54.6]	1.000
	No	55	131	186	29.6 [23.5–36.5]	
Family atopy	Yes	57	121	178	32.0 [25.6–39.2]	0.039^b^
	No	2	20	22	9.1 [2.5–27.8]	

95% confidence intervals calculated using Wilson's method.

a Pearson's chi-square global test.

b Fisher's exact test

### Management

All children with a positive SPT received a cow's milk protein elimination diet and regular allergology follow-up. Those with negative tests were clinically monitored without specific dietary restriction.

## Discussion

This retrospective study conducted in the Immunoallergology Unit of the University Hospital of Cocody, Abidjan, Côte d’Ivoire identified a 29.5% prevalence of IgE sensitisation to cow's milk proteins among children under 3 years of age referred for suspected allergy. This rate, comparable to values reported in specialised settings (20–35%),^6^^,^^12^ should be interpreted with caution because of the selection bias inherent in hospital-based recruitment. In the general population, sensitisation rates are much lower: a community-based study in Cameroon reported a prevalence of only 1.4%,^8^ underscoring the impact of recruitment context on the estimation of allergic sensitisation.

Cutaneous manifestations predominated, particularly eczema, which is often regarded as an early marker of the “atopic march”.^1^ Although eczema was the most frequently reported cutaneous symptom in the medical records, the analysis was performed on a composite “cutaneous signs” variable due to inconsistent distinction between eczema and urticaria. The association between eczema and IgE sensitisation is well established; studies have shown that skin inflammation promotes loss of tolerance and the production of specific IgE antibodies.^13^ In our cohort, both eczema and a family history of atopy were significantly associated with a positive SPT, confirming their predictive value in clinical assessment.^14^^,^^15^ Conversely, digestive symptoms were less frequently linked to sensitisation, consistent with observations by Nachshon and Jeong,^2^^,^^16^ suggesting that isolated gastrointestinal presentations often correspond to non-IgE-mediated forms.

From a pathophysiological perspective, IgE-mediated reactions follow the classical cascade of immediate hypersensitivity: antigen presentation, Th2 differentiation, production of allergen-specific IgE, binding to mast cells, and degranulation upon re-exposure.^17^ This mechanism explains the diagnostic relevance of the SPT, which reproduces an in-vivo local allergic response and remains a reliable first-line tool in infancy.^1^^,^^6^^,^^18^ Recent work has highlighted the role of milk-specific T-cell epitopes in sustaining IgE responses in some children,^19^ suggesting that distinct immunological profiles may underlie the variable clinical evolution.

In resource-limited countries such as those in sub-Saharan Africa, the SPT remains an accessible and effective test for initial diagnosis. Limited availability of specific IgE assays or OFCs represents a major constraint, supporting the use of a stepwise diagnostic strategy based on clinical and skin-test data.^20^ Nevertheless, a multidimensional approach combining SPT with serological assays, when feasible, is recommended by international guidelines^7^^,^^21^ to enhance diagnostic specificity and reduce unnecessary dietary exclusions. A comparison of SPT results obtained with commercial extracts versus the prick-to-prick technique was not feasible, as the choice of method depended on reagent availability and was not systematically recorded.

Therapeutically, most children with a positive SPT received a cow's-milk elimination diet, consistent with the recommendations of the World Allergy Organization (WAO) and European Society for Paediatric Gastroenterology Hepatology and Nutrition (ESPGHAN).^7^^,^^22^ However, restricted access to hypoallergenic formulas, either extensively hydrolysed or amino acid-based, remains a major challenge across Africa. Amino acid-based formulas have demonstrated efficacy not only for symptom control but also in promoting tolerance development,^23^ an essential component of early desensitisation strategies. Locally available plant-based substitutes such as soy, rice, or millet preparations are sometimes used but require rigorous nutritional and allergological evaluation before being incorporated into national care protocols. In this retrospective study, potential adverse reactions to these nutritional substitutes could not be assessed, as this information was not consistently documented in the medical records.

Finally, practice variability between centres highlights the need for standardised protocols for the management of paediatric CMPA. A recent multicentre comparison^24^ showed that strategies integrating close clinical correlation and periodic reassessment significantly reduced the risk of unjustified prolonged elimination. Implementing a simplified decision pathway based on suggestive symptoms, family atopy, and SPT results would constitute a context-appropriate approach.

### Limitations

This study has several limitations. First, its retrospective design entails selection bias, since included children were those referred for suspected allergy and therefore do not represent the general population. Variability in medical record quality may also have introduced heterogeneity in the clinical data collected. Moreover, no documented cases of anaphylaxis were identified in the available records, preventing any analysis of severe IgE-mediated presentations. Second, the absence of longitudinal follow-up prevents assessment of clinical evolution among sensitised children, including spontaneous resolution or persistence of sensitisation. Third, diagnosis relied solely on the SPT, without confirmation by specific IgE measurement or OFC. This may have led to over- or under-estimation of the true rate of clinical allergy and prevented assessment of the clinical relevance of positive SPTs, as the proportion of true cow's milk allergy among sensitised children could not be determined. Finally, potentially relevant variables, such as the precise diversification pattern, age at introduction of cow's milk proteins, or the exact type of milk used, could not be analysed comprehensively due to missing data.

## Conclusion

This study revealed a notable proportion of IgE-mediated sensitisation to cow's milk proteins among children under 3 years of age referred for suspected allergy. In resource-limited settings, the skin prick test represents a reliable and accessible tool for the initial triage of suspected CMPA. Identifying suggestive clinical profiles, particularly early eczema and a family history of atopy, can help prioritise children who require specialist follow-up. Strengthening local capacity and training in paediatric allergy care, together with the implementation of standardised diagnostic and nutritional follow-up protocols, should be prioritised to optimise CMPA management and reduce unnecessary dietary restrictions in sub-Saharan Africa.

## Consent to participate

Not applicable. This retrospective study used anonymised data from routine clinical records with no direct patient involvement.

## Data availability

Individual-level data are not available owing to the retrospective nature of the study and the absence of electronic records. All aggregated data used for analysis are fully presented in the tables of the manuscript.

## Author contributions

**Goran-Kouacou APV**: Conceptualization; Investigation; Data curation; Writing-Original Draft; Writing-Review and Editing.

**Adou AH**: Methodology; Validation; Formal analysis; Writing-Review and Editing.

**Séri YJ, Yéboah OR, Mondah A:** Data collection; Records extraction and verification; Investigation; Writing-Review and Editing.

**Assi AUA, Niamba NI**: Data curation; Database management; Resources; Writing-Review and Editing.

**N'Guessan K, Siransy KL**: Supervision; Writing-Review and Editing.

**Dassé SR**: Conceptualization; Supervision; Project administration; Writing-Review and Editing.

All authors approved the final version of the manuscript.

## Ethics approval

The study received ethical approval from the Ethics Committee of the Société Ivoirienne d’Hématologie, d’Immunologie et d’Oncologie - Transfusion Sanguine (SIHIO-TS) under reference number N°47_2025-SIHIOTS. This retrospective analysis used anonymised data extracted from routine clinical records. No direct patient contact occurred, and no identifiable information was collected. The study was conducted in accordance with the principles of the Declaration of Helsinki.

## Authors’ consent for publication

All authors consent to the publication of this manuscript.

## AI disclosure

DeepL Translator (DeepL SE, Cologne, Germany), an AI-assisted translation tool, was used for language editing and translation.

## Funding

The authors declare that they received no financial support for the conduct of this study.

## Competing interests

The authors declare no conflicts of interest.
